# Mitochondria-targeted cyclosporin A delivery system to treat myocardial ischemia reperfusion injury of rats

**DOI:** 10.1186/s12951-019-0451-9

**Published:** 2019-01-25

**Authors:** Chang-xiong Zhang, Ying Cheng, Dao-zhou Liu, Miao Liu, Han Cui, Bang-le Zhang, Qi-bing Mei, Si-yuan Zhou

**Affiliations:** Department of Pharmaceutics, School of Pharmacy, Fourth Military Medical University, Changle West Road 169, Xi’an, 710032 Shaanxi China

**Keywords:** Myocardial ischemia/reperfusion injury, Mitochondrial targeting, Cyclosporin A, Mitochondrial permeability transition pore, Mitochondria-targeted peptide

## Abstract

**Background:**

Cyclosporin A (CsA) is a promising therapeutic drug for myocardial ischemia reperfusion injury (MI/RI) because of its definite inhibition to the opening of mitochondrial permeability transition pore (mPTP). However, the application of cyclosporin A to treat MI/RI is limited due to its immunosuppressive effect to other normal organ and tissues. SS31 represents a novel mitochondria-targeted peptide which can guide drug to accumulate into mitochondria. In this paper, mitochondria-targeted nanoparticles (CsA@PLGA-PEG-SS31) were prepared to precisely deliver cyclosporin A into mitochondria of ischemic cardiomyocytes to treat MI/RI.

**Results:**

CsA@PLGA-PEG-SS31 was prepared by nanoprecipitation. CsA@PLGA-PEG-SS31 showed small particle size (~ 50 nm) and positive charge due to the modification of SS31 on the surface of nanoparticles. CsA@PLGA-PEG-SS31 was stable for more than 30 days and displayed a biphasic drug release pattern. The in vitro results showed that the intracellular uptake of CsA@PLGA-PEG-SS31 was significantly enhanced in hypoxia reoxygenation (H/R) injured H9c2 cells. CsA@PLGA-PEG-SS31 delivered CsA into mitochondria of H/R injured H9c2 cells and subsequently increased the viability of H/R injured H9c2 cell through inhibiting the opening of mPTP and production of reactive oxygen species. In vivo results showed that CsA@PLGA-PEG-SS31 accumulated in ischemic myocardium of MI/RI rat heart. Apoptosis of cardiomyocyte was alleviated in MI/RI rats treated with CsA@PLGA-PEG-SS31, which resulted in the myocardial salvage and improvement of cardiac function. Besides, CsA@PLGA-PEG-SS31 protected myocardium from damage by reducing the recruitment of inflammatory cells and maintaining the integrity of mitochondrial function in MI/RI rats.

**Conclusion:**

CsA@PLGA-PEG-SS31 exhibited significant cardioprotective effects against MI/RI in rats hearts through protecting mitochondrial integrity, decreasing apoptosis of cardiomyocytes and myocardial infract area. Thus, CsA@PLGA-PEG-SS31 offered a promising therapeutic method for patients with acute myocardial infarction.

**Electronic supplementary material:**

The online version of this article (10.1186/s12951-019-0451-9) contains supplementary material, which is available to authorized users.

## Background

Ischemic heart disease is a leading cause of death and disability worldwide [1], and early reperfusion with thrombolytic therapy or percutaneous transluminal coronary intervention has been showed to be effective in reducing myocardial infarct size and improving cardiac function. However, clinical trials indicate that the efficacy of adjunctive therapies in reducing myocardial infarct size is not satisfied [2, 3]. The researches show that morbidity and mortality remain substantial in patients with ST-segment elevation acute myocardial infarction (STEMI). 1 year after myocardial ischemia, the mortality and the incidence of heart failure is 7% and 22%, respectively [4]. The phenomenon indicates the process of myocardial reperfusion can induce further cardiomyocyte death, which is known as myocardial ischemia reperfusion injury (MI/RI).

Although the exact mechanism of MI/RI is still not very clear at present, the previous experimental studies have identified that the opening of mitochondrial permeability transition pore (mPTP) is one of the key factors for MI/RI. mPTP is a non-selective and high-conductance channel which locates in the inner mitochondrial membrane [5]. The mPTP remains closed conformation during ischemia and open conformation after myocardial reperfusion. mPTP opening induces the collapse of mitochondrial membrane potential, efflux of cytochrome c, breakdown of ATP production, and ultimately, death of cardiomyocyte. Therefore, the opening of mPTP is one of the important mechanisms that lead to reperfusion injury and cardiomyocyte death [6, 7].

It was reported that the opening of the mPTP could be inhibited by the immunosuppressive agent cyclosporin A (CsA) [8]. CsA inhibits mPTP opening by binding with cyclophilin D (CypD) that locates in the inner mitochondrial membrane [9–13]. Creatine kinase (CK) release reduced 40% over 72 h in a small cohort of patients with acute STEMI when they received 2.5 mg/kg CsA (iv) at 10 min before carrying out primary percutaneous coronary intervention (PCI) with direct stenting [14]. CsA (2.5 mg/kg, iv, 10 min before aortic unclamping) also decreased accumulative troponin I release in patients during aortic valve surgery [15]. However, CsA is a systemic immunosuppressant. After CsA is systemically administered, it leads to immunosuppressive effects in organs that CsA are distributed. In addition, CsA exerts protective effects on MI/RI only when CsA is delivered into the inner mitochondrial membrane of ischemic cardiomyocyte. Thus, in theory, targeted delivery of CsA to the mitochondria of ischemic cardiomyocyte during reperfusion can improve its protective effects on MI/RI and attenuate its systemic immunosuppressive effects at the same time. However, at present, targeted delivery of CsA to mitochondria of ischemic cardiomyocyte is rare in spite of the significant roles of mPTP in the MI/RI. Therefore, it is highly desirable to specifically deliver CsA into mitochondria of ischemic cardiomyocyte.

The enhanced permeability and retention (EPR) effect in ischemic myocardium is the same as that in tumor tissues [16–20]. Thus, CsA-loaded PLGA nanoparticles can concentrate at the myocardial tissue of ischemia reperfusion through the EPR effect [21]. However, PLGA nanoparticle can be non-specifically uptaken by the mononuclear phagocyte system (MPS) in the blood circulation due to its lipophilicity [22, 23], which reduces the accumulation of CsA-loaded PLGA nanoparticle in mitochondria of ischemic cardiomyocyte. More seriously, when CsA-loaded PLGA nanoparticle has been uptaken by MPS, it will attenuate the activity of MPS. Subsequently, it will decrease the immune function of the whole body. Fortunately, the hydrophilicity of PLGA nanoparticle can be markedly increased by coating polyethylene glycol (PEG) on PLGA nanoparticle surface, which subsequently decreases the nonspecific uptake by MPS [24, 25]. This will consequently result in a long blood circulation time of PLGA nanoparticle and an increase of accumulation of PLGA nanoparticle into the mitochondria of ischemic cardiomyocyte.

The SS31 (Szeto-Schiller 31) peptide specifically concentrates on the inner mitochondrial membrane through interacting with cardiolipin without depending mitochondrial membrane potential [26]. This is a unique advantage for mitochondria-targeted drug delivery of ischemic cardiomyocyte because mitochondrial membrane potential is damaged during ischemia reperfusion. Thus, SS31 represents a novel mitochondria-targeted peptide to guide drug to accumulate into the mitochondria of ischemic cardiomyocyte.

Therefore, in this study, we prepared SS31 and PEG modified PLGA nanoparticle (CsA@PLGA-PEG-SS31) to precisely deliver CsA to mitochondria of ischemic myocardium. The uptake and intracellular distribution of nanoparticles on hypoxia reoxygenation (H/R) injured H9c2 cells were investigated for illuminating the role of SS31. Then the efficacy and its mechanism of CsA@PLGA-PEG-SS31 on MI/RI were deeply evaluated through in vitro and in vivo experiments.

## Results

### Characterization of CsA@PLGA-PEG-SS31

**Fig. 1:**
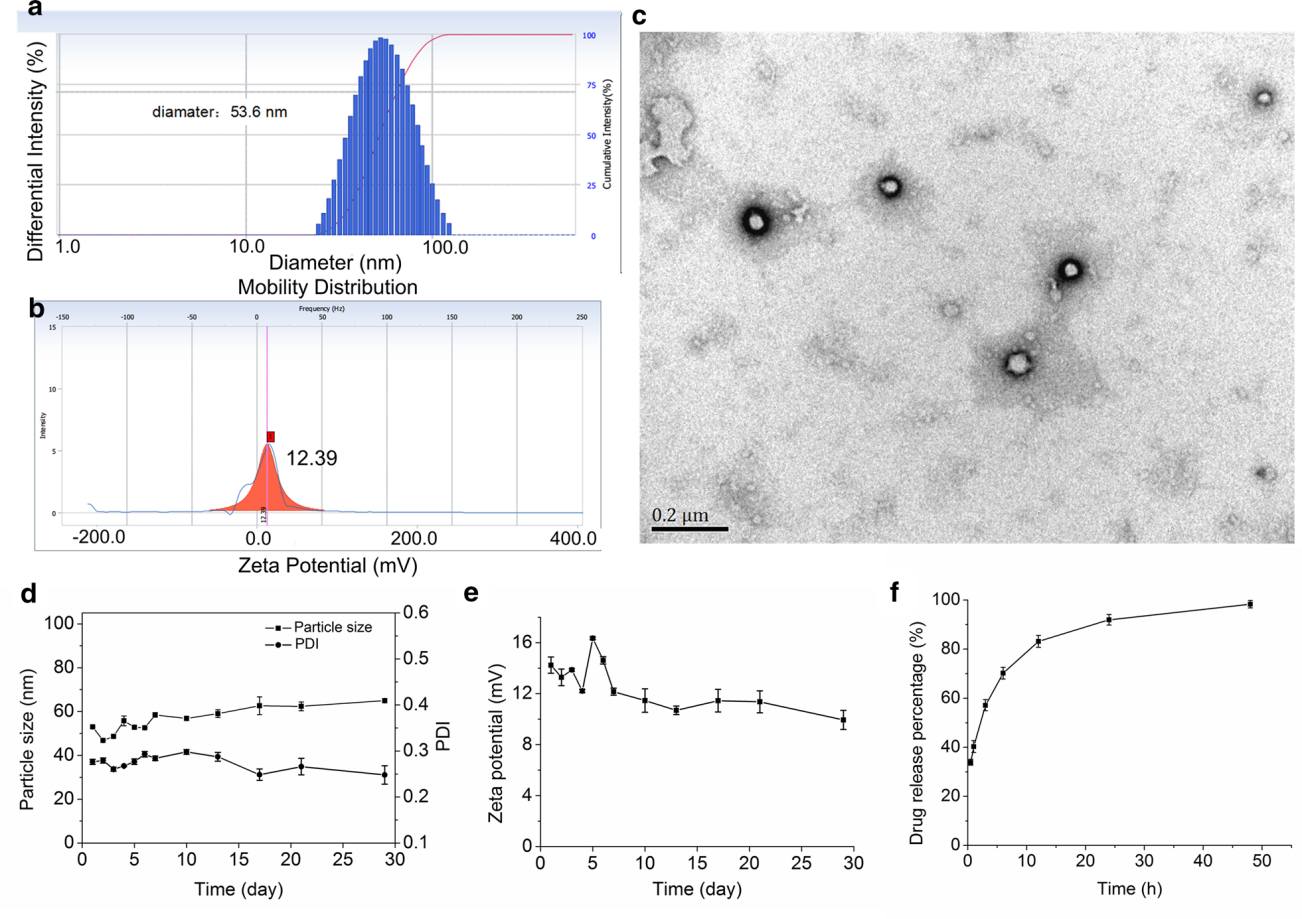
Physicochemical and in vitro characterization of CsA@PLGA-PEG-SS31. **a** Size distribution of CsA@PLGA-PEG-SS31. **b** Zeta potential of CsA@PLGA-PEG-SS31. **c** TEM image of CsA@PLGA-PEG-SS31. **d** The particle size of CsA@PLGA-PEG-SS31 in deionized water. **e** The zeta potential of CsA@PLGA-PEG-SS31 in deionized water. **f** In vitro drug release profiles of CsA@PLGA-PEG-SS31 in 40% ethanol

The drug loading of CsA@PLGA-PEG-SS31 was 3.5%. As showed in Fig. 1a–c, the average hydrated diameter of the CsA@PLGA-PEG-SS31 was 54 nm, and polydispersity index (PDI) value was 0.18. Due to the SS31 moiety on the surface of nanoparticle, the zeta potential of CsA@PLGA-PEG-SS31 was + 14.5 mV. The TEM images showed that the nanoparticles were spherical in shape and uniformly distributed without aggregation.

### Stability and drug release profiles of CsA@PLGA-PEG-SS31

The stability of CsA@PLGA-PEG-SS31 in deionized water is showed in Fig. 1d, e. The results indicated that CsA@PLGA-PEG-SS31 was stable for more than 30 days. This was resulted from the repulsive effect between the positive charged nanoparticles. After 30 days, the stability was gradually lost, which might be due to the degradation of PLGA-PEG-SS31. To illuminate the drug release profile of CsA@PLGA-PEG-SS31, 40% ethanol was used as the release medium. The release characteristic from CsA@PLGA-PEG-SS31 is showed in Fig. 1f, and CsA@PLGA-PEG-SS31 displayed a biphasic drug release pattern. The drug release rate was fast in the initial period (at the first 1 h), and followed by a slow and sustained release. Approximately 90% of CsA was released in 24 h, and all loaded drugs were released in 48 h.

### Hemolysis effect of CsA@PLGA-PEG-SS31

Due to the positive charge of CsA@PLGA-PEG-SS31, hemolysis experiment was used to reflect the in vivo safety of CsA@PLGA-PEG-SS31 after intravenous injection [27]. The hemolytic effect of blank nanoparticles on rat red cells is showed in Additional file 1: Table S1. The hemolysis rate of the blank nanoparticles exhibited dose-dependent manners. The hemolysis rate of the blank nanoparticles was less than 5% when their concentration ranged from 1 mg/mL to 2 mg/mL. In addition, the cytotoxicity of blank nanoparticle (@PLGA-PEG-SS31 and @PLGA-PEG) on H9c2 cell is showed in Additional file 1: Figure S1. @PLGA-PEG-SS31 and @PLGA-PEG did not show significant cytotoxicity when their concentration ranged from 0 to 3700 μg/mL. All above data implied the biocompatibility of CsA@PLGA-PEG-SS31 when it was intravenous injected. Moreover, when concentration of CsA in CsA@PLGA-PEG-SS31 was higher than 50 μg/mL, CsA@PLGA-PEG-SS31 showed significant toxicity on H9c2 cells. Thus, the protective effect of CsA@PLGA-PEG-SS31 on H9c2 cell was investigated at the dose that less than 30 μg/mL.

### Protective effect of CsA@PLGA-PEG-SS31 on hypoxia reoxygenation (H/R) injured H9c2 cells

**Fig. 2:**
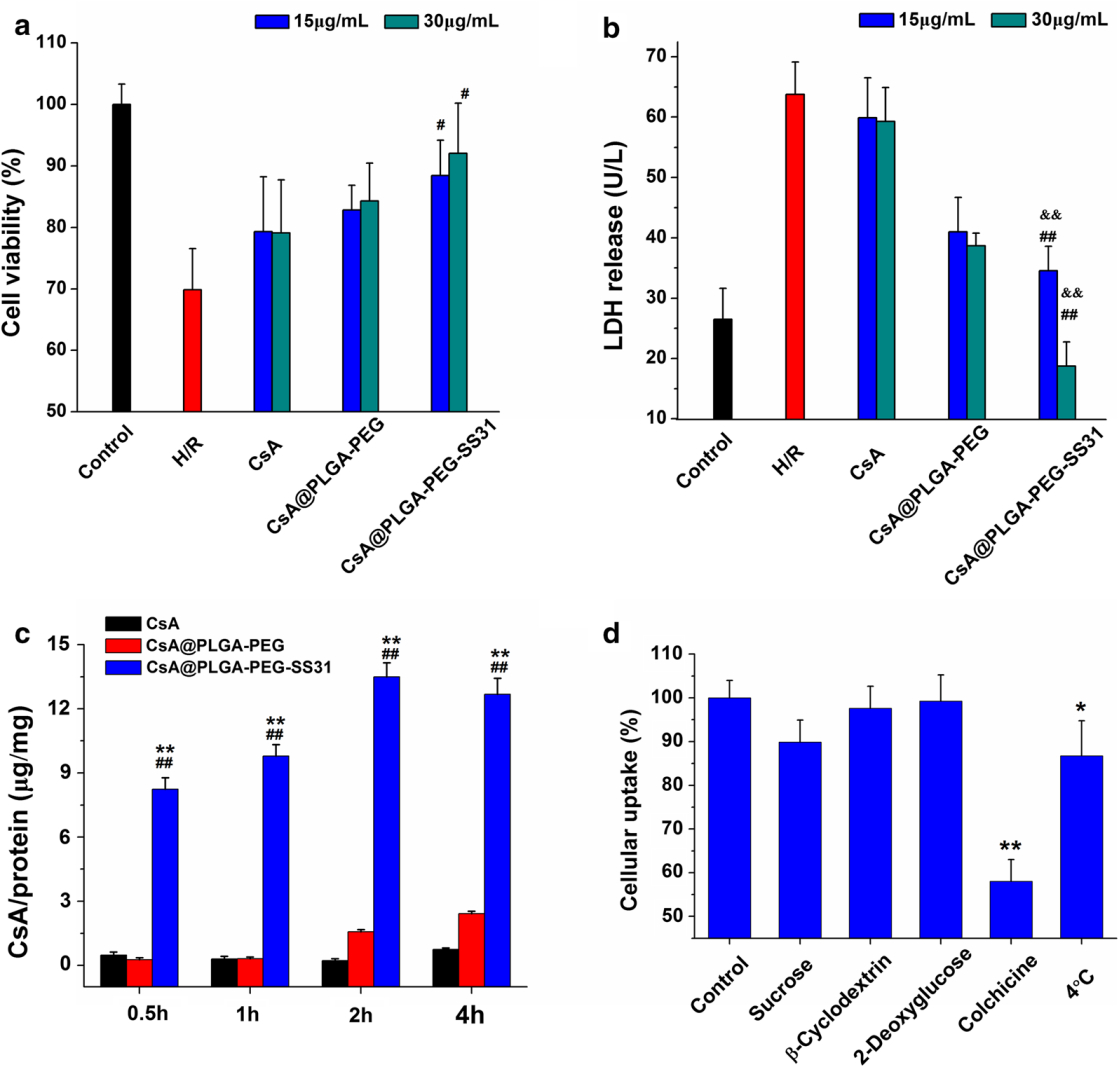
**a** Cell viability of H/R injury H9c2 cell after treatment with different concentration of CsA, CsA@PLGA-PEG and CsA@PLGA-PEG-SS31. ^#^p < 0.05 versus CsA group at the same concentration. **b** LDH release from H/R injury H9c2 cell after treatment with different concentration of CsA, CsA@PLGA-PEG and CsA@PLGA-PEG-SS31. ^##^p < 0.01 versus CsA group at the same concentration, ^&&^p < 0.01 versus CsA@PLGA-PEG group at the same concentration. **c** Cellular uptake of CsA, CsA@PLGA-PEG and CsA@PLGA-PEG-SS31 in H/R injured H9c2 cells (determined by HPLC). ^##^p < 0.01 versus CsA group at the same time point, **p < 0.01 versus CsA@PLGA-PEG group at the same time point. **d** Relative uptake efficiency of CsA@PLGA-PEG-SS31 on H/R injured H9c2 cells at the presence of various endocytosis inhibitors, ATP depletion or 4 °C. **p < 0.01, *p < 0.05 versus control group

The protective effect of CsA@PLGA-PEG-SS31 on hypoxia reoxygenation (H/R) injured H9c2 cells is showed in Fig. 2a. Hypoxia for 3 h and reoxygenation for 4 h led to the obvious decrease of viability of H9c2 cell, whereas pretreatment with CsA, CsA@PLGA-PEG and CsA@PLGA-PEG-SS31 increased viability of H/R injured H9c2 cells in dose-dependent manner. CsA@PLGA-PEG-SS31 exerted significant protective effect as compared with CsA and CsA@PLGA-PEG. Lactic dehydrogenase (LDH) is a biomarker of cardiomyocyte damage. LDH was released in cell culture medium after the H9c2 cells were injured by H/R. As showed in Fig. 2b, LDH release markedly increased in H/R group as compared with that in control group. CsA, CsA@PLGA-PEG and CsA@PLGA-PEG-SS31 obviously decreased the LDH release in dose-dependent manner. CsA@PLGA-PEG-SS31 exhibited strongest inhibition effect on LDH release. The above data indicated that CsA@PLGA-PEG-SS31 could protect H9c2 cells from H/R-induced injury.

### The cellular uptake of CsA@PLGA-PEG-SS31 in H/R injured H9c2 cells

The cellular uptake of CsA@PLGA-PEG-SS31 in H/R injured H9c2 cells is showed in Fig. 2c. The uptake of CsA, CsA@PLGA-PEG and CsA@PLGA-PEG-SS31 in H/R injured H9c2 cells displayed time-dependent manner, and the uptake of CsA@PLGA-PEG-SS31 reached equilibrium in 2 h. CsA@PLGA-PEG-SS31 significantly increased the uptake of CsA by H/R injured H9c2 cells. Figure 2d showed that colchicine obviously inhibited the cellular uptake of CsA@PLGA-PEG-SS31. This suggested that CsA@PLGA-PEG-SS31 was internalized by H/R injured H9c2 cells mainly through macropinocytosis pathways.

### Mitochondrial delivery of CsA by CsA@PLGA-PEG-SS31

**Fig. 3:**
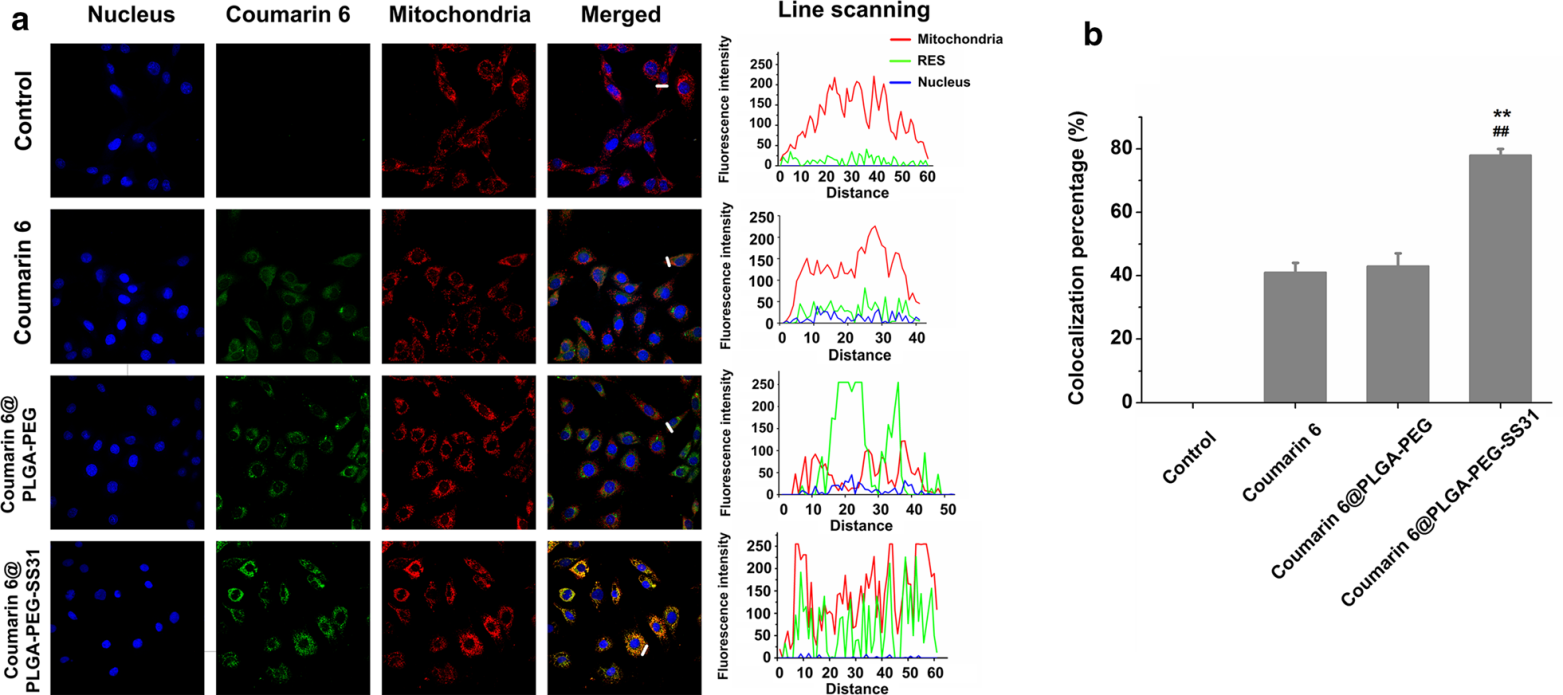
The intracellular trafficking of CsA@PLGA-PEG-SS31 in H/R injured H9c2 cells. **a** The mitochondrial distribution and the fluorescence intensity after free coumarin 6, coumarin 6 labeled CsA@PLGA-PEG (coumarin 6@PLGA-PEG), and coumarin 6 labeled CsA@PLGA-PEG-SS31 (coumarin 6@PLGA-PEG-SS31) were incubated with H/R injured H9c2 cells for 4 h (60 × 10). The fluorescence intensity was graphed according to the white line of merged fluorescence images. **b** Colocalization efficiency of coumarin 6 after free coumarin 6, coumarin 6 labeled CsA@PLGA-PEG (coumarin 6@PLGA-PEG) and coumarin 6 labeled CsA@PLGA-PEG-SS31 (coumarin 6@PLGA-PEG-SS31) incubated with H/R injured H9c2 cells for 4 h (×60). **p < 0.01 versus coumarin 6 group, ^##^p < 0.01 versus coumarin 6@PLGA-PEG group

As showed in Fig. 3a, after H/R injured H9c2 cells were cultured with coumarin 6 labeled CsA@PLGA-PEG-SS31 for 4 h, the merged CLSM images showed the overlap between coumarin 6 green fluorescence and MitoTracker red fluorescence. This indicated that a large amount of coumarin 6 was delivered into mitochondria. In contrast, after H/R injured H9c2 cells were cultured with coumarin 6 labeled CsA@PLGA-PEG for 4 h, the merged images of H/R injured H9c2 cells displayed slightly yellow color, which indicated little amount of coumarin 6 was delivered into mitochondria. In addition, the colocalization efficiency of coumarin 6 and mitochondria is showed in Fig. 3b. The colocalization efficiency was 78%, 43% and 41% for coumarin 6 labeled CsA@PLGA-PEG-SS31, coumarin 6 labeled CsA@PLGA-PEG and free coumarin 6, respectively. The above data indicated that the SS31 moiety played an important role in delivery CsA into mitochondria.

### The effect of CsA@PLGA-PEG-SS31 on H/R-induced mitochondrial injury in H9c2 cells

**Fig. 4:**
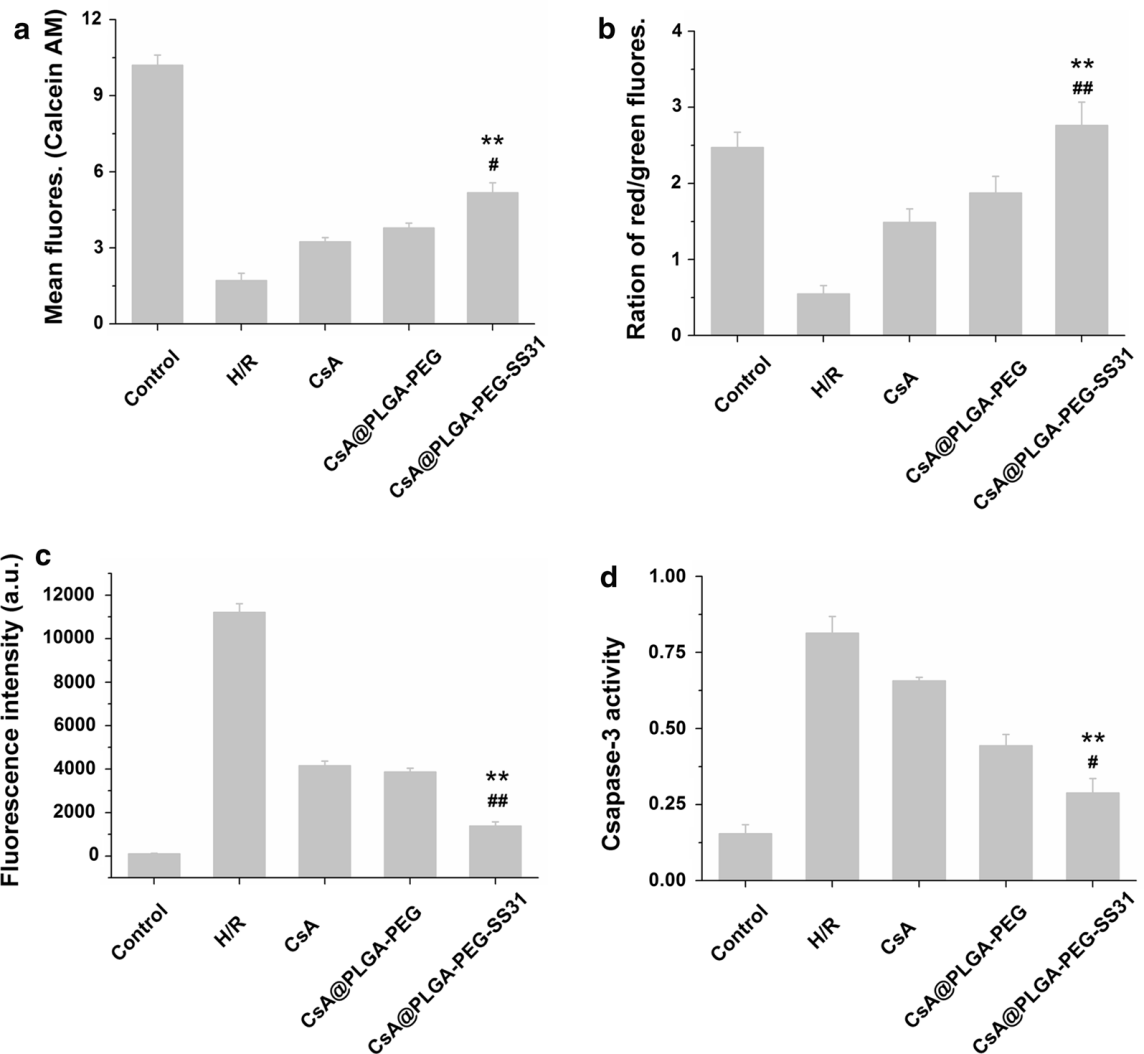
The protective effects of CsA@PLGA-PEG-SS31 on H/R injured H9c2 cells. **a** Calcien AM fluorescence intensity measured in H/R injured H9c2 cells by flow cytometry. **b** The ratio of red/green fluorescence intensity measured by fluoro-spectrophotometer in H/R injured H9c2 cells after JC-1staining. **c** MitoSOX fluorescence intensity measured in H/R injured H9c2 cells by flow cytometry. **d** Caspase 3 activity measured by fluoro-spectrophotometer in H/R injured H9c2 cells. **p < 0.01 versus CsA group; ^#^p < 0.05, ^##^p < 0.01 versus CsA@PLGA-PEG group

The effect of CsA@PLGA-PEG-SS31 on mPTP opening in H/R injured H9c2 cells is showed in Fig. 4a. The fluorescence intensity in H/R injured H9c2 cells was significantly reduced as compared with that in normal group, indicating that the opening of mPTP in H/R injured H9c2 cells increased. When H/R injured H9c2 cells were pretreated with CsA, CsA@PLGA-PEG and CsA@PLGA-PEG-SS31, fluorescence intensity in H/R injured H9c2 cells was enhanced. This implied the mPTP opening was inhibited by CsA, CsA@PLGA-PEG and CsA@PLGA-PEG-SS3. Besides, the results also indicated that CsA@PLGA-PEG-SS31 exerted the strongest inhibition for mPTP opening in H/R injured H9c2 cells. Opening of mPTP in the inner mitochondrial membrane results in the collapses of the membrane potential (∆Ψ_m_). The changes of ∆Ψ_m_ are showed in Fig. 4b. The normal cells exhibited high ratio between red fluorescence intensity and green fluorescence intensity. However, the ration of red/green in H/R injured H9c2 cells decreased significantly. After pretreatment with CsA, CsA@PLGA-PEG and CsA@PLGA-PEG-SS31, the ratio of red/green fluorescence increased, demonstrating a recovery of ∆Ψm. CsA@PLGA-PEG-SS31 significantly increased ∆Ψm as compared with CsA and CsA@PLGA-PEG. Because a large amount of CsA was delivered into mitochondria by CsA@PLGA-PEG-SS31, consequently CsA@PLGA-PEG-SS31 could significantly protect mitochondrion through inhibiting the collapses of ∆Ψm.

Reactive oxygen species (ROS) level in mitochondria is closely related to the integrity of mitochondrial inner membrane. The effect of CsA@PLGA-PEG-SS31 on mitochondrial ROS is showed in Fig. 4c. The fluorescence intensity in H/R injured H9c2 cells was significantly increased as compared with that in normal group, which indicated that a large amount of ROS was produced in mitochondria in H/R injured H9c2 cells. When H/R injured H9c2 cells were pretreated with CsA, CsA@PLGA-PEG and CsA@PLGA-PEG-SS31, the fluorescence intensity significantly decreased. This indicated CsA, CsA@PLGA-PEG and CsA@PLGA-PEG-SS31 reduced the ROS levels in mitochondria. Moreover, the results also indicated CsA@PLGA-PEG-SS31 exhibited the strongest inhibitive effect on the production of mitochondrial ROS.

### The effect of CsA@PLGA-PEG-SS31 on the cleaved caspase-3 level in H/R injured H9c2 cells

The effect of CsA@PLGA-PEG-SS31 on the cleaved caspase-3 level in H/R injured H9c2 cells is showed in Fig. 4d. When H/R injured H9c2 cells were cultured with CsA, CsA@PLGA-PEG and CsA@PLGA-PEG-SS31, the cleaved caspase-3 level in H/R injured H9c2 cells was decreased. Beside, CsA@PLGA-PEG-SS31 markedly decreased the caspase-3 activity in H/R injured H9c2 cells. This implied that CsA@PLGA-PEG-SS31 could markedly reduce the apoptosis of H/R injured H9c2 cells.

### The accumulation of CsA@PLGA-PEG-SS31 in ischemic myocardium

**Fig. 5:**
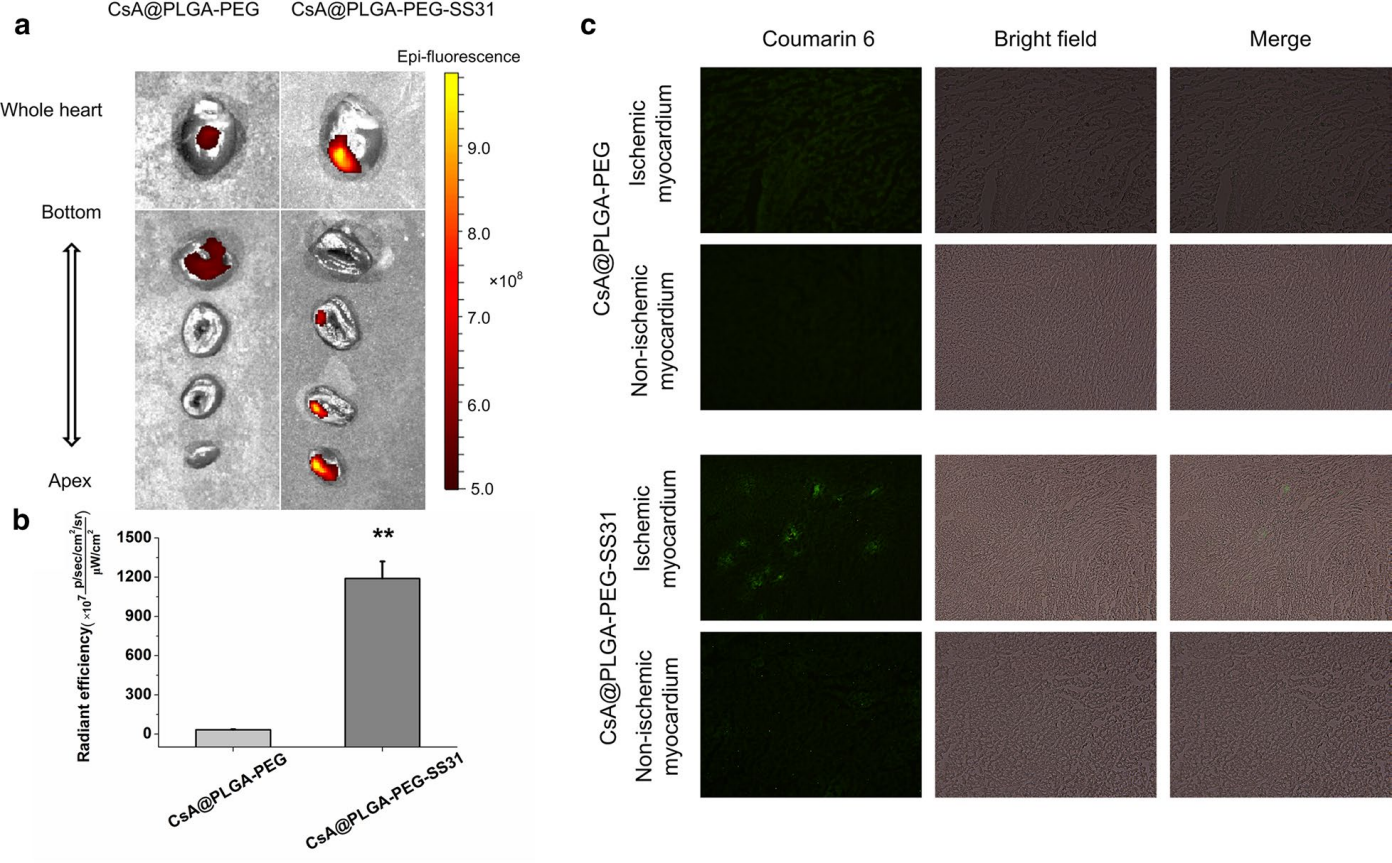
Representative ex vivo fluorescence images (**a**) and quantitative analysis (**b**) of cy7.5 labelled CsA@PLGA-PEG and cy7.5 labelled CsA@PLGA-PEG-SS31 in MI/RI rat heart 1 h post-injection. **c** Representative fluorescence microscope image of coumarin 6 labelled CsA@PLGA-PEG and coumarin 6 labelled CsA@PLGA-PEG-SS31 in ischemic myocardium tissue and normal myocardium tissue 2 h post-injection. **p < 0.01 versus CsA@PLGA-PEG group

The accumulation of cy7.5 labeled CsA@PLGA-PEG-SS31 in ischemic myocardium was visualized by using in vivo imaging technology, and the result is showed in Fig. 5a, b. Compared with cy7.5 labeled CsA@PLGA-PEG, more cy7.5 labeled CsA@PLGA-PEG-SS31 was accumulated in ischemic tissue in MI/RI rat heart at 1 h post-injection. Besides, the distribution of cy7.5 labeled CsA@PLGA-PEG-SS31 and cy7.5 labeled CsA@PLGA-PEG in 2 mm thickness section of left ventricle was also observed by using in vivo imaging technology, and more amount of cy7.5 labeled CsA@PLGA-PEG-SS31 accumulated in cardiac apex as compared with cy7.5 labeled CsA@PLGA-PEG. Furthermore, 2 h after injection of nanoparticle, the distribution of coumarin 6 labeled CsA@PLGA-PEG-SS31 and coumarin 6 labeled CsA@PLGA-PEG in 5 μm thickness slice of ischemic myocardium tissue was observed by using fluorescence microscope, and the result is showed in Fig. 5c. After coumarin 6 labeled CsA@PLGA-PEG-SS31 was intravenously injected to normal rats and MI/RI rats, more amount of coumarin 6 labeled CsA@PLGA-PEG-SS31 was found in the slice of ischemic myocardium tissue of MI/RI rats, while little amount of coumarin 6 labeled CsA@PLGA-PEG-SS31 was found in the slice of normal myocardium tissue of MI/RI rats. Compared with coumarin 6 labeled CsA@PLGA-PEG-SS31, less amount of coumarin 6 labeled CsA@PLGA-PEG was found in the slice of ischemic myocardium tissue of MI/RI rats. The above results demonstrated that CsA@PLGA-PEG-SS31 could accumulate in the ischemic tissue and penetrate into more deep area of ischemic myocardium.

### The protection of CsA@PLGA-PEG-SS31 on heart function of MI/RI rats

**Fig. 6:**
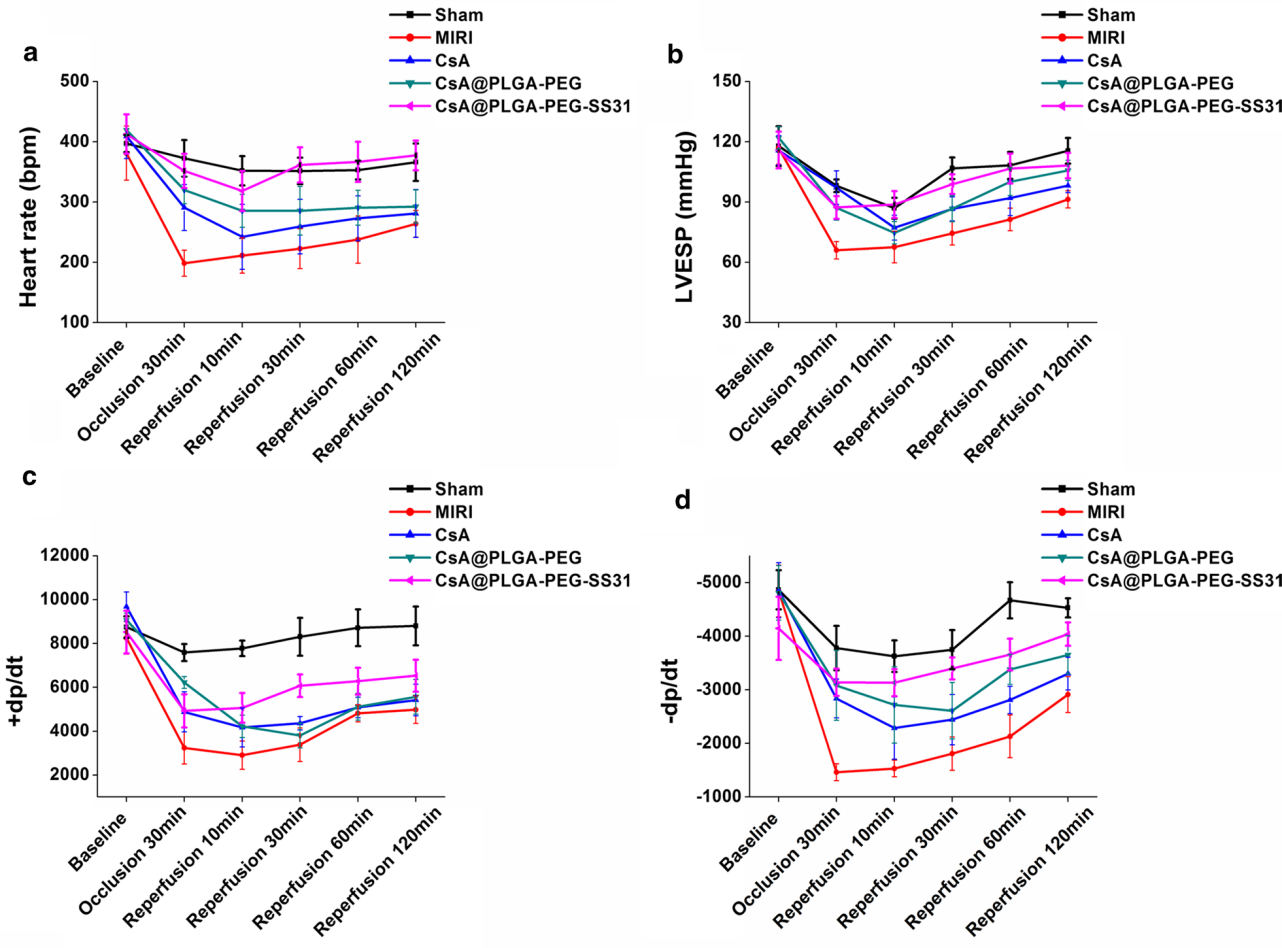
Heart rate (**a**), left ventricular end-systolic pressure (LVESP) (**b**), + dp/dt_max_ (**c**), and − dp/dt_max_ (**d**) during ischemia and reperfusion in rats

Parameters of left ventricular function were recorded and calculated at the 30 min of ischemia and 2 h of reperfusion in MI/RI rats.  Figure 6a–d showed that ischemia/reperfusion (IR) significantly decreased  ± dp/dt_max_, LVESP and heart rate as compared with sham group. Pretreatment with CsA, CsA@PLGA-PEG and CsA@PLGA-PEG-SS31 attenuated IR-induced cardiac dysfunction. At the same time, CsA@PLGA-PEG-SS31 showed strongest protective effect in terms of heart rate and  ± dp/dt_max_. This manifested CsA@PLGA-PEG-SS31 obviously increased the cardiac function of MI/RI rats.

**Fig. 7:**
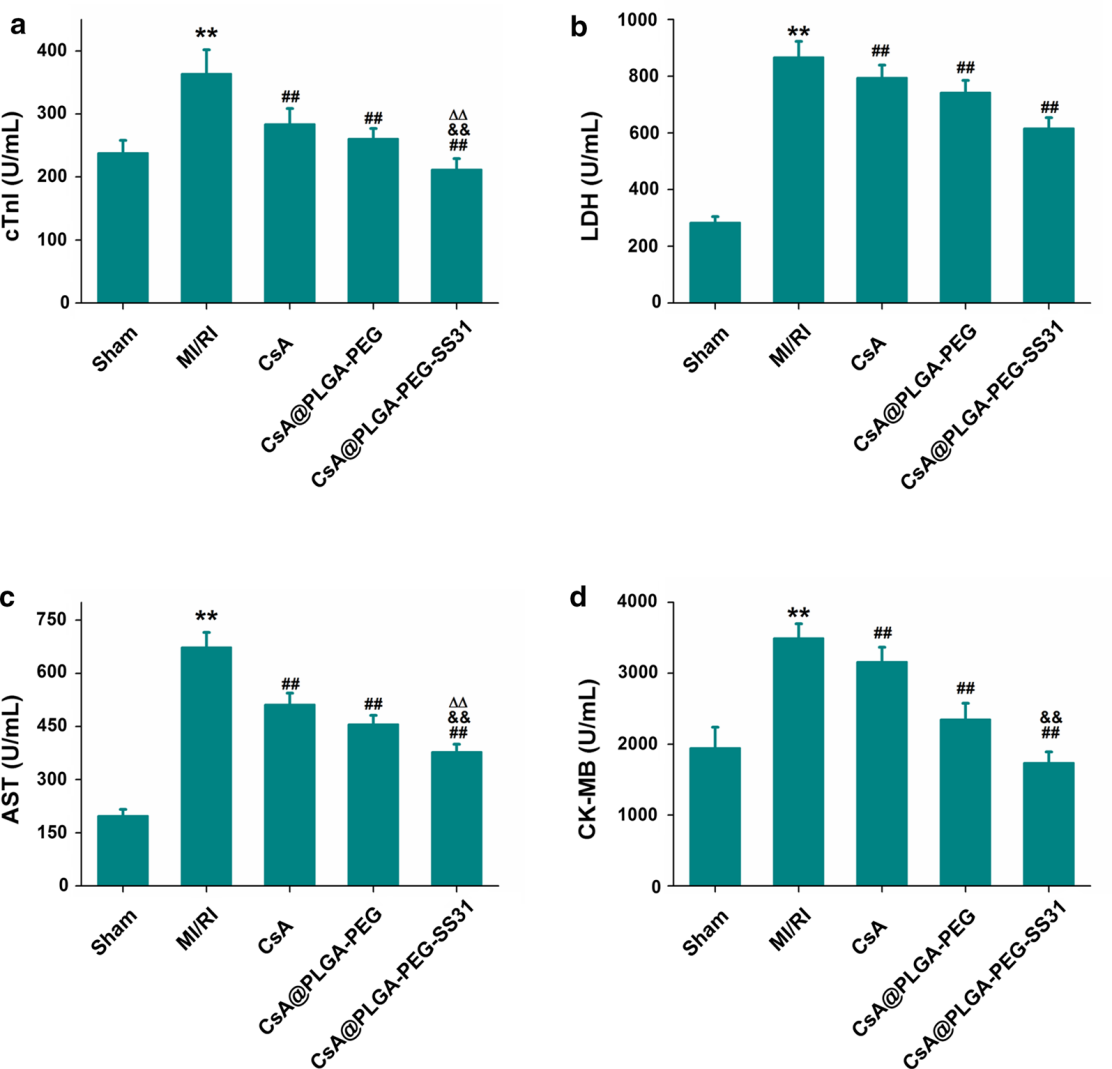
The level of cTnI (**a**), LDH (**b**), AST (**c**) and CK-MB (**d**) in plasma at the end of reperfusion. **p < 0.01 versus sham group; ^##^p < 0.01 versus MI/RI group; ^△△^p < 0.01 versus CsA group; ^&&^p < 0.01 versus CsA@PLGA-PEG group

In clinic, the level of LDH, CK-MB, AST and cTnI in serum was used to assess the extent of myocardial damage. As showed in Fig. 7, CK-MB, LDH, AST and cTnI were obviously reduced in CsA, CsA@PLGA-PEG and CsA@PLGA-PEG-SS31 group. CsA@PLGA-PEG-SS31 showed the strongest inhibitive effect on the release of CK-MB, LDH, AST and cTnI. This indicated that myocardial damage was obviously attenuated through pretreatment of CsA@PLGA-PEG-SS31.

**Fig. 8:**
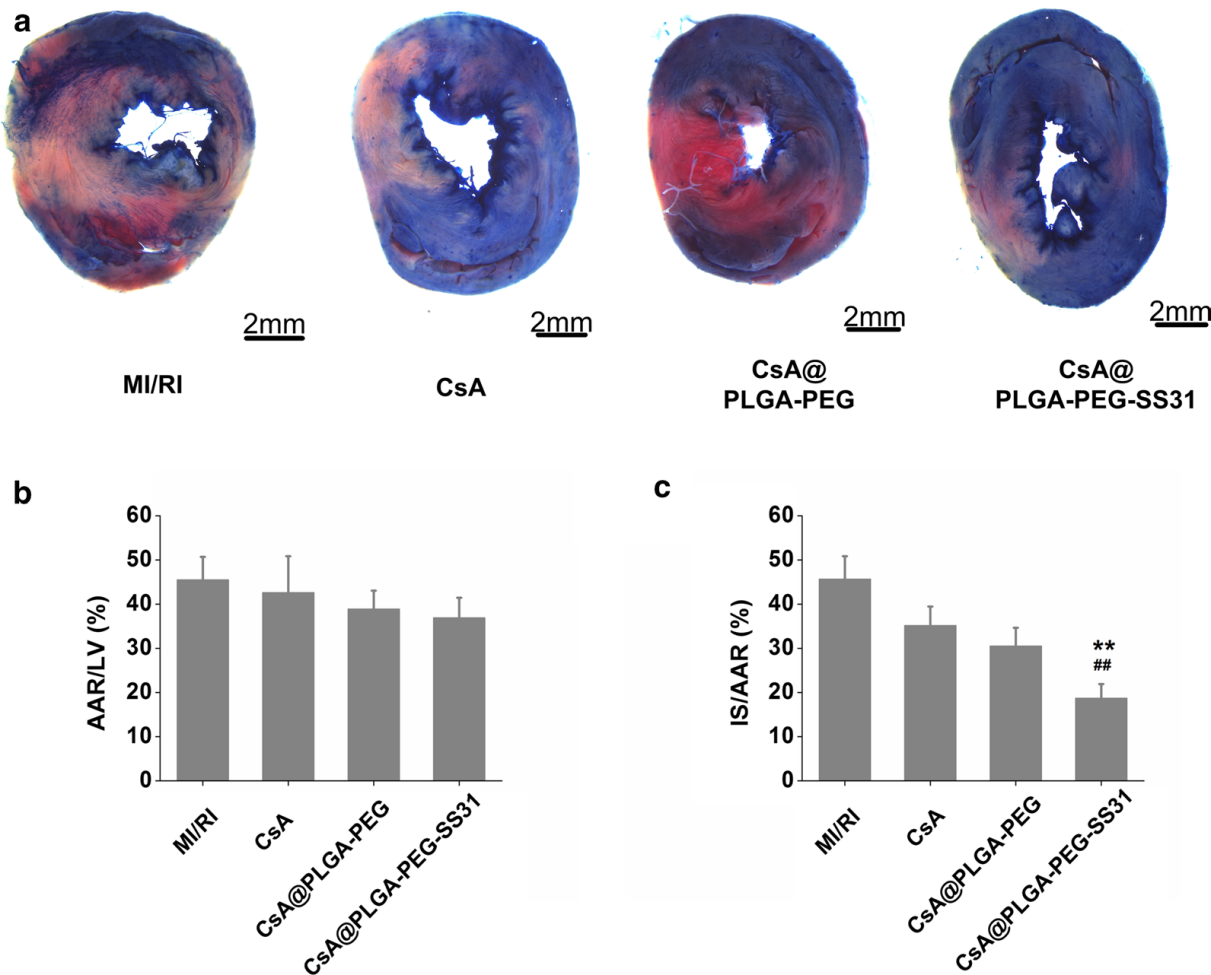
**a** Evans Blue and 2,3,5-triphenyltetrazolium chloride (TTC) staining of myocardium. The blue area indicates non-ischemic area. The red area indicates viable tissue at risk area. The white area indicates infarct size; **b** quantitative analysis of ratio of area at risk to area of left ventricle (AAR/LV); **c** quantitative analysis of the percentage of infarct size (IS) in area at risk (IS/AAR). **p < 0.01 versus CsA group; ^##^p < 0.01 versus CsA@PLGA-PEG group

The infarct size is a direct index of myocardial injury. The TTC/evans blue staining of heart tissue are showed in Fig. 8. There was no significant difference in AAR/LV between MI/RI group and pretreatment group. This ensured the consistency of injury in different groups. The percentage of infarct area was 46% in MI/RI group, whereas the percentage of infarct area was 19% in CsA@PLGA-PEG-SS31 pretreatment group, indicating the obvious cardioprotective effect of CsA@PLGA-PEG-SS31 during IR process. Those data also demonstrated that CsA@PLGA-PEG-SS31 protected cardic function by reducing the infarct size in MI/RI rats.

### The effect of CsA@PLGA-PEG-SS31 on histomorphology of heart tissue

**Fig. 9:**
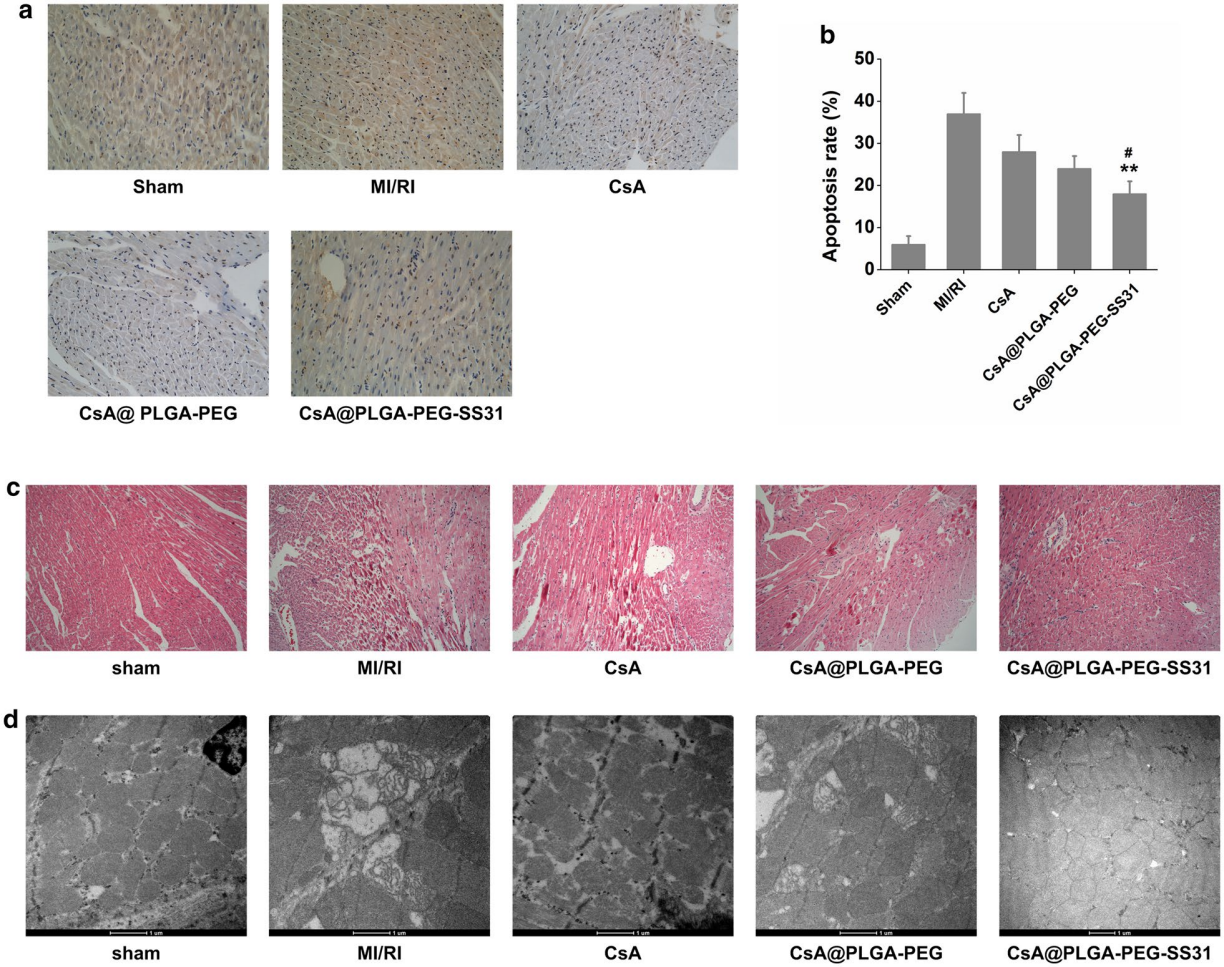
**a** Representative photomicrographs of TUNEL staining of myocardium section. Nucleus was stained with DAPI (blue), and apoptotic nucleus was detected by TUNEL staining (brown). **b** Quantitative analysis of the ratio of TUNEL positive nucleus to DAPI-stained nucleus. ^∗∗^p < 0.01 versus CsA group. ^#^< 0.05 versus CsA@PLGA-PEG group. **c** Histopathological changes of representative myocardium sections; **d** representative TEM images of mitochondria of myocardium sections, Scale bar = 500 nm

Representative photographs of TUNEL staining of heart section are showed in Fig. 9a, b. TUNEL-positive cells (brown color) were rarely found in the sham group, and a number of TUNEL-positive cell were observed in MI/RI group. CsA, CsA@PLGA-PEG and CsA@PLGA-PEG-SS31 significantly reduced the number of TUNEL-positive cell. This implied pretreatment of CsA, CsA@PLGA-PEG and CsA@PLGA-PEG-SS31 attenuated cardiomyocyte apoptosis. Moreover, the apoptosis was significantly alleviated in rat heart when MI/RI rats were pretreated with CsA@PLGA-PEG-SS31.

Histopathological changes in ischemic myocardium in MI/RI rats were assessed by H&E staining, and the results are showed in Fig. 9c. In MI/RI group, heart tissues exhibited a lot of necrosis area, myocardial structural disarray, and a large number of inflammatory cells. The histological features of heart tissue became almost normal in CsA@PLGA-PEG-SS31 group. Mitochondria were the key target organelle of MI/RI, and mitochondrial damage is closely related to apoptosis of cardiomyocytes. Thus, TEM was used to observe the mitochondrial morphology of ischemic myocardium in MI/RI rats, and the results are showed in Fig. 9d. In MI/RI group, the mitochondria of cardiomyocyte showed swell, disappearance of cristae and vacuolization. However, CsA@PLGA-PEG-SS31 group showed more cristae structures and less vacuolar shape. The above data indicated that CsA@PLGA-PEG-SS31 protected myocardium from damage by reducing the recruitment of inflammatory cells and maintaining the integrity of mitochondrial function in MI/RI rats.

## Discussion

In the present study, a mitochondria-targeted PLGA nanoparticle was prepared to deliver CsA to mitochondria of ischemic myocardium. CsA@PLGA-PEG-SS31 displayed a biphasic drug release pattern in vitro. The rapid release in the initial stage was resulted from the release of the drug adsorbed on the surface of nanoparticles and drug wrapped around the outlayer of nanoparticles, and this was of great benefit to the rapid recovery of the acute damage of cardiomyocyte. The sustained release was caused by the drug diffusion from nanoparticle and the lower drug concentration gradient between the CsA@PLGA-PEG-SS31 and the release medium [28], and this was advantageous to the continuous treatment of MI/RI. Besides, the results of hemolytic experiment stated that blank nanoparticle did not induce any significant hemolysis when their concentration ranged from 1 to 2 mg/mL. According to the American Society for Testing and Materials (ASTM F756-00, 2000), a material should be classified as having non-hemolytic effect if the hemolysis rate is less than 5% [29]. This implied that CsA@PLGA-PEG-SS31 was safe for intravenous injection.

Triphenylphosphine (TPP) is a commonly used moiety that guides nanoparticle to accumulate into mitochondria. The targeting effect of TPP depends on the mitochondrial membrane potential. However, mitochondrial membrane potential is collapsed in MI/RI. SS31 can specifically concentrate in the inner mitochondrial membrane through interacting with cardiolipin without depending on mitochondrial membrane potential. In addition, SS31 showed significant efficacy in reducing oxidative stress, especially in experimental models of myocardial ischemia [26, 30]. Therefore, SS31 is an ideal mitochondrial targeting moiety. The experimental results indicated that more amount of CsA was delivered into mitochondria of H/R inured H9c2 cells by CsA@PLGA-PEG-SS31 as compared with that by CsA@PLGA-PEG. This implied that SS31 played a very important role in delivering CsA into mitochondria.

The results of the uptake experiments showed that the CsA@PLGA-PEG-SS31 could significantly increase cellular uptake of CsA by H/R inured H9c2 cells. The remarkable increase in cellular uptake was related to the modification of SS31. It was reported that the internalization rate of positively charged 50 nm nanoparticle by cardiomyocyte was 3-fold higher than that of the same-sized nanoparticle with a negative charge [31]. Thus, the positive charge arisen from SS31 was one important contributor to the high uptake of CsA@PLGA-PEG-SS31 by H/R injured H9c2 cells. Besides, SS31 showed cell-permeable effect [32], which also contributed to the high uptake efficiency of CsA@PLGA-PEG-SS31 in H/R injured H9c2 cells.

mPTP opening can cause severe damage on mitochondrial integrity, which dramatically decreases mitochondrial function and results in the release of cytochrome c from mitochondria, the activation of caspase 3, and finally the apoptosis of cardiomyocyte. CsA@PLGA-PEG-SS31 exhibited stronger inhibitive effect on mPTP opening and collapse of mitochondrial membrane potential in H/R injured H9c2 cells. At the same time, CsA@PLGA-PEG-SS31 decreased ROS level and cleaved caspase 3 activity in H/R injured H9c2 cells. The above results were the consequence of that more amount of CsA was delivered into the mitochondria of H/R injured H9c2 cells by CsA@PLGA-PEG-SS31.

The application of CsA to treat MI/RI is limited due to the immunosuppression of CsA to other normal organ and tissues. In addition, CsA can exert protective effects on MI/RI only when CsA is delivered into the inner mitochondrial membrane of ischemic cardiomyocyte. CsA@PLGA-PEG-SS31 quickly accumulated in MI/RI heart due to the EPR effect of ischemia myocardium [33]. Furthermore, CsA@PLGA-PEG-SS31 could permeate the ischemic heart tissue more deeply, which resulted from the membrane penetrating property and positive charge of SS31 [32].

Haemodynamics are the important indicators of cardiac function. CsA@PLGA-PEG-SS31 increased the ± dp/dt_max_, LVESP and heart rate. Besides, CsA@PLGA-PEG-SS31 decreased the release of CK-MB, LDH, AST and cTnI from MI/RI-injured myocardium. All of the above data indicated that CsA@PLGA-PEG-SS31 exhibited obviously cardioprotective effects against MI/RI in rats. Furthermore, apoptosis of cardiomyocyte is a critical factor of MI/RI-induced cardiac dysfunction [34]. It is widely considered that the decrease of cardiomyocytic apoptosis can attenuate the loss of cardiomyocytes and finally reduce or even prevent the occurrence and progress of MI/RI [35]. Mitochondria play an important role in cardiomyocyte apoptosis. The experimental results indicated that CsA@PLGA-PEG-SS31 decreased the opening of mPTP and maintained the integrity and function of mitochondria, which subsequently decreased the cardiomyocyte apoptosis of MI/RI rat and the infarct size of MI/RI rat. All those beneficial effects finally improved the cardic function of MI/RI rat.

## Conclusion

CsA@PLGA-PEG-SS31 delivered a large amount of CsA into mitochondria of H/R injured cardiomyocyte. CsA@PLGA-PEG-SS31 increased the cell viability and decreased the LDH release through inhibiting mPTP opening, mitochondrial membrane potential collapses and cleaved caspase-3 level in H/R injured H9c2 cells. In addition, CsA@PLGA-PEG-SS-31 delivered more amount of CsA into ischemic myocardium, consequently, CsA@PLGA-PEG-SS31 exhibited cardioprotective effects against MI/RI in rats through protecting mitochondrial integrity, decreasing cardiomyocyte apoptosis and myocardial infraction area. In a word, CsA@PLGA-PEG-SS-31 showed great potential in the treatment of acute myocardial infarction.

## Materials and methods

### Materials

Cyclosporine A (CsA) was purchased from Shanghai Yuanye Biotechnology Co., Ltd (Shanghai, China). PLGA-PEG-SS31 was bought from Chinese Peptide Co., Ltd (Hangzhou, China. The mass spectrum of SS31-PEG8 is showed in Additional file 1: Figure S2. The ^1^H NMR spectrum of SS31-PEG8-PLGA is showed in Additional file 1: Figure S3). The lactate dehydrogenase assay kit was purchased from Nanjing Jiancheng Bioengineering Institute (Nanjing, China). The mitochondrial membrane potential test kit (JC-1 solution), cleaved caspase 3 activity test kit, active oxygen test kit, tissue mitochondrial isolation kit, and RIPA lysate were purchased from Nanjing Beyotime Biotechnology Co. (Nanjing, China). Mitochondrial red fluorescent probe (Mito-tracker deep red FM), mitochondrial superoxide red fluorescent probe (MitoSOX red mitochondrial superoxide indicator), and mitochondrial membrane permeability transition hole detection kit were purchased from Shanghai Yisheng Biotechnology Co. (Shanghai, China). H9c2 cell line (cardiomyocyte of rat) was bought from Shanghai Institute of Cell Biology (Shanghai, China). SD rats were provided by the Experimental Animal Center of the Fourth Military Medical University (Xi’an, China).

### Preparation and characterization of CsA@PLGA-PEG-SS31

The nanoprecipitation method was used to prepare CsA@PLGA-PEG-SS31. In brief, 3 mg CsA and 12 mg PLGA-PEG-SS31 were dissolved in 4 mL acetone and was used as the oil phase, 10 mL deionized water was used as the aqueous phase. Then oil phase was slowly added to the water phase under stirring. The emulsion was then stirred for 6 h until acetone is completely evaporated at room temperature. The colloid was filtered through 0.4 μm filter membrane. PLGA-PEG was used to prepare CsA@PLGA-PEG. CsA@PLGA-PEG-SS31 solution was placed in a cuvette, and the zeta potential and particle size of CsA@PLGA-PEG-SS31 were determined by using dynamic light scattering (DLS, Beckman Coulter Particle Analyzer, Fullerton, California, US). The morphology of CsA@PLGA-PEG-SS31 was observed by using transmission electron microscopy (TEM, JEOL-100CXII, Japan). The drug release behavior of CsA@PLGA-PEG-SS31 was evaluated by using the dialysis method. The CsA concentration was measured by high performance liquid chromatograph (HPLC, Waters 2695/2996, Massachusetts, USA). The regression equation of the standard curve was y = 656.78x − 542.02 (R^2^ = 0.9996) when CsA concentration ranged from 1 to 100.0 μg/mL.

In order to determine whether the blank nanoparticle @PLGA-PEG-SS31 can cause hemolysis, red blood cells of rat were used to perform the hemolysis test. After being washed with PBS for three times, erythrocytes were dispersed in PBS (pH 7.4) and incubated with @PLGA-PEG-SS31 or @PLGA-PEG (1 mg/mL and 2 mg/mL). Deionized water and normal saline were used as positive and negative control, respectively. The erythrocyte mixture solution was incubated at 37 °C for 40 min and centrifuged at 750*g* for 5 min. The supernatant was collected and the mixture of ethanol (99%, v/v) and hydrochloric acid (37%, w/v) (39:1) was added. The absorbance (OD value) was detected by using spectrophotometry at 398 nm. The hemolysis ratio (HR %) was calculated as the following equation: HR (%) = [(OD_sample_ − OD_negative_)/(OD_positive_ − OD_negative_)] × 100%.

### The protective effect of CsA@PLGA-PEG-SS31 on hypoxia reoxygenation injured H9c2 cells

Sodium chloride (4.007 g), potassium chloride (0.59 g), magnesium chloride (0.05 g), hydrated calcium chloride (0.065 g), 4-hydroxyethylpiperazine ethane sulfonic acid (0.475 g), 2-deoxy-d-glucose (0.82 g), sodium sulfate (0.093 g) and sodium lactate (1.12 g) were added to 500 mL of deionized water to prepare hypoxic solution.

The hypoxia reoxygenation (H/R) injured H9c2 cells model was established to imitate the heart ischemia reperfusion injury. H9c2 cells were incubated with hypoxic culture medium for 3 h in a hypoxic environment (95% N_2_ and 5% CO_2_) at 37 °C. Then, the hypoxic culture medium was removed and DMEM without fetal bovine serum (FBS) was added. H9c2 cells were cultured for 4 h in a standard incubator with 5% CO_2_ in normal atmosphere at 37 °C. Drug treatment was carried out at the beginning of reoxygenation. The control group was exposed to normoxic conditions with DMEM without FBS for 7 h.

MTT assay and LDH release were used to investigate the protective effect of CsA@PLGA-PEG-SS31 on H/R injured H9c2 cells. H9c2 cells were seeded in 96-well plates (1 × 10^4^ cells/well) and cultured for 48 h. After that, the cells were incubated in hypoxic environment for 3 h, then DMEM containing CsA, CsA@PLGA-PEG or CsA@PLGA-PEG-SS31 was added to the wells. After cells were incubated for 4 h, 20 μL of culture medium was collected to test the release of lactic dehydrogenase (LDH) by using lacate dehydrogenase assay kit (Nanjing Jiancheng Bioengineering Institute, China). Then 5 mg/mL of MTT (20 μL) was added to the 96-well plate and then the plate was put in incubator. After 4 h, the formazan crystals in the plate were solubilized with 150 μL DMSO, and the absorbance of DMSO solution at 490 nm was quantified by a microplate reader (Bio-Rad Laboratories, Richmond, CA, USA).

### Cellular uptake of CsA@PLGA-PEG-SS31

H9c2 cells were seeded into 6 well plates (1 × 10^5^ cells/well). After hypoxia for 3 h, cells were incubated with DMEM containing CsA, CsA@PLGA-PEG or CsA@PLGA-PEG-SS31 (30 μg CsA/mL) for 0.5 h, 1 h, 2 h and 4 h, respectively. Cells were washed for 3 times with PBS (pH 7.4) and lysed by 100 μL RIPA lysis buffer. To investigate the endocytic pathway of CsA@PLGA-PEG-SS31, 2-deoxy-d-glucosesucrose (ATP depletion, 1 mg/mL), sucrose (inhibitor of clathrin-mediated uptake, 150 mg/mL), methyl-β-cyclodextrin (inhibitor of caveolae-mediated uptake, 0.005 mg/mL), colchicine (inhibitor of macropinocytosis, 0.8 mg/mL) were respectively added to H/R injured H9c2 cells, and the cells were incubated for 1 h at 37 °C in hypoxic culture medium. Then, the cells were cultured with fresh DMEM containing CsA, CsA@PLGA-PEG or CsA@PLGA-PEG-SS31 (30 μg CsA/mL) and incubated for 2 h. The cells were collected and lysed. Finally, CsA in cell lysis was determined by HPLC. The protein content in cell lysis was determined by coomassie brilliant blue. The CsA in cell lysis was normalized by protein content in cell lysis.

### Mitochondrial distribution of CsA delivered by CsA@PLGA-PEG-SS31

Coumarin 6 labeled CsA@PLGA-PEG-SS31 was used to investigate the subcellular distribution of CsA delivered by CsA@PLGA-PEG-SS31. H9c2 cells were planted into cover glass-containing 24 well plates (5 × 10^4^ cells/well) and were cultured for 24 h. After hypoxia for 3 h, the cells were cultured with fresh DMEM containing coumarin 6 labeled CsA@PLGA-PEG or CsA@PLGA-PEG-SS31 for 4 h. Cells were incubated with MitoTracker green (100 nmol/L) for 30 min. After that, the cells were fixed with 4% paraformaldehyde. Finally, the cells were incubated with DAPI solution (100 μg/mL) for 15 min. The distribution of coumarin 6 labeled nanoparticle in the cells was observed by confocal laser screen microscopy (CLSM). All CLSM images were obtained in the sequential parameter setting to make the co-localization study more convincing.

### Determination of mitochondrial membrane potential

H9c2 cells were planted in a 6 well plate (2 × 10^5^ cells per well) and cultured for 48 h. After hypoxia for 3 h, hypoxic solution was replaced by fresh DMEM containing CsA, CsA@PLGA-PEG or CsA@PLGA-PEG-SS31 (30 μg CsA/mL) for 4 h, and then the cells were incubated with JC-1 solution (5 μg/mL) for 15 min at 37 °C and washed three times with assay buffer. After collecting the cells, the red fluorescence intensity of cell solution at 530/590 nm (excitation/emission wavelength) and green fluorescent intensity of cell solution at 485/530 nm were detected by fluorescence spectrophotometer. The ratio of red to green fluorescent intensity of each sample was calculated [36].

### Determination of mPTP opening

Calcein AM and CoCl_2_ were used to detect mitochondrial permeability transition pore opening. H9c2 cells were planted in a 6 well plate (2 × 10^5^ cells per well) and cultured for 48 h. After hypoxia for 3 h, the cells were cultured with fresh DMEM containing CsA, CsA@PLGA-PEG or CsA@PLGA-PEG-SS31 (30 μg CsA/mL) for 4 h, and then the cells were collected and incubated with 5 μL Calcein AM solution (1 mmol/L) and 5 μL CoCl_2_ solution (80 mmol/L) for 15 min at 37 °C. After that, 3.5 mL of HBSS/Ca was added into the tubes and cells were collected through centrifugation. Finally, the cells were resuspended in 400 μL buffer for flow cytometric analysis [37].

### Determination of cleaved caspase-3 level

The effect of CsA@PLGA-PEG-SS31 on the cleaved caspase-3 level in H/R injured H9c2 cells was detected by using cleaved caspase 3 activity assay kit (Beyotime Institute of Biotechnology, Nanjing, China). Briefly, H9c2 cells were seeded in 6 well plate for 48 h. After the hypoxia for 3 h, the cells were cultured with fresh DMEM containing CsA, CsA@PLGA-PEG or CsA@PLGA-PEG-SS31 (30 μg CsA/mL) for 4 h. Then the cells were collected and resuspended in RIPA buffer for lysing. After centrifuging for 15 min at 4 °C, the supernatant was collected. At last, 10 μL Ac-DEVD-pNA (2 mmol/L) and 40 μL supernatant were added into a 6 well plate. After incubating for 1 h, the absorbance of the solution at 405 nm was quantified by a microplate reader (Bio-Rad Laboratories, Richmond, CA, USA) [38].

### Distribution of CsA@PLGA-PEG-SS31 in rat hearts after MI/RI

To establish the rat myocardium ischemia/reperfusion injury model, male Sprague–Dawley rats were anesthetized with pentobarbital sodium at a dose of 60 mg/kg. The heart was exposed through a thoracotomy performed in the fourth or fifth intercostal space. The suture thread was used to occlude the left anterior descending coronary artery (LAD) for 30 min through a small piece of polyethylene tubing to serve as a reversible snare occluding. 5 min before reperfusion, Cy7.5-labeled CsA@PLGA-PEG-SS31 (or coumarin 6 labeled CsA@PLGA-PEG-SS31) and Cy7.5-labeled CsA@PLGA-PEG (or coumarin 6 labeled CsA@PLGA-PEG) was respectively administered through the tail vein at a dose of 2.5 mg/kg. After 1 h, the heart was isolated and perfused with PBS for 2 min to wash out blood from heart tissue. The distribution of the Cy7.5-labeled CsA@PLGA-PEG-SS31 and Cy7.5-labeled CsA@PLGA-PEG was visualized and analyzed by using in vivo image (Caliper IVIS Lumina II, Caliper Life Science, USA). In addition, the heart was cut into 2 mm thickness section to clearly observe location of the Cy7.5-labeled CsA@PLGA-PEG-SS31 and Cy7.5-labeled CsA@PLGA-PEG in the heart tissue by using in vivo image. Finally, 2 h after injection of nanoparticle, ischemic myocardium tissue and normal myocardium tissue were cut into 5 μm thickness slice, and the distribution of coumarin 6 labeled CsA@PLGA-PEG-SS31 and coumarin 6 labeled CsA@PLGA-PEG in slices was observed by using fluorescence microscope.

### Measurement of myocardial infarct size

Evans Blue/TTC dual staining was applied to determine the myocardial infarct size as previously described [39]. 2 h after reperfusion, the suture thread around the coronary artery was retied, and then 1 mL Evans Blue solution (2%) was injected into the aorta. After 2 min, the heart was quickly removed and washed with PBS before being frozen at − 20 °C. After freezing for 24 h, the heart was cut into 5 slices (about 2 mm thickness) and incubated with 1% TTC for 10 min at 37 °C in the dark conditions. The normal myocardium was stained into deep blue by Evans Blue. TTC colored myocardium at risk but still viable red. Infarcted myocardium appeared pale after TTC staining. Areas of infarct size (IS) and area at risk (AAR) were determined digitally by Image-Pro Plus 7.0 (Media Cybernetics, Bethesda, MD, USA). The percentage of infarct size was quantified as (IS/AAR) × 100% in a blind manner, and percentage of AAR was expressed as (AAR/LV) × 100%.

### Histopathologic examination of ischemic myocardium

After the blood sample was collected, the rat heart was removed. Heart tissue was fixed in 10% paraformaldehyde, then embedded in paraffin. Finally, the tissue was cut into 5 μm thickness of slice. The slice was stained with hematoxylin and eosin (H&E). The histopathological changes were observed by optical microscope.

### Tunel staining of ischemic myocardium

TUNEL assay kit (terminal transferase-mediated d UTP-fluorescein nick end labeling) was used to stain the apoptotic cells in the heart tissue sample. The slices were observed by using Axioscope fluorescence microscope. Percentage of apoptosis (%) = (total number of apoptotic cells/total number of cells) × 100%.

### Measurement of LDH, CK-MB, AST and cTnI in serum

Myocardial damage was evaluated by measuring plasma concentration of LDH, CK-MB, AST and cTnI. At the end of the experiment, blood was collected and serum was separated by centrifugation. The LDH, CK-MB and AST were detected by biochemical analyzer. Troponin I (cTnI) was detected by using the ELISA Assay Kit.

### Determination of heart function after MI/RI

During ischemia and reperfusion, the maximum increase rate of left ventricular pressure (+ dp/dt_max_) and the maximum decrease rate of left ventricular pressure (− dp/dt_max_), heart rate (HR) and left ventricular end-systolic pressure (LVESP) were recorded by using a computerized powerlab (California, USA).

### Mitochondrial morphology in ischemic myocardium

After 2 h of reperfusion in rats, the heart was removed and the anterior left ventricular tissue below the ligature 2 mm was clipped and washed once with cold PBS. After that, the tissue was placed in glutaraldehyde (4%) to be preserved at 4 °C for 48 h. Then, the tissue was incubated with osmic acid for 4 h before being embedded in epoxy resin. After polymerizing at 60 °C, the embedded samples were cut into 70 nm thickness slices. 4% uranyl acetate/4% lead citrate was used to stain the slice. Transmission electron microscope was used to observe the morphology of mitochondria after the slice was put on a cooper grid.

### Statistical analysis

All data presented in this article was expressed as mean ± SD and the statistical analysis of the data was performed by Student’s t-test. For all comparisons, p < 0.05 was considered significant (SPSS 22.0).

## Additional file

**Additional file 1.** Additional figures and table.

## Abbreviations

AST: aspartate aminotransferase
CK-MB: creatine kinase MB
CLSM: confocal laser scanning microscopy
cTnI: troponin I
+dp/dt: the rate of the rise of the left ventricular pressure
H&E: hematoxylin and eosin
HR: heart rate
LDH: lactic dehydrogenase
LVESP: left ventricular end systolic pressure
ΔΨm: mitochondrial membrane potential
MI/RI: myocardial ischemia reperfusion injury
mPTP: mitochondrial permeability transition pore
MTT: methyl thiazolyl tetrazolium
PEG: polyethylene glycol
PLGA: poly(d,l-lactide-co-glycolide)
SS31: Szeto-Schiller peptide 31
TUNEL: terminal-deoxynucleoitidyl transferase mediated nick end labeling
