# Supplementary material for: Mitochondria-targeted cyclosporin A delivery system to treat myocardial ischemia reperfusion injury of rats
Source: J Nanobiotechnology. 2019 Jan 25;17:18. doi: 10.1186/s12951-019-0451-9 (PMC6346555; doi:10.1186/s12951-019-0451-9)
Supplement: Supplementary file 1 — Additional file 1. Additional figures and table. [file 12951_2019_451_MOESM1_ESM.docx]

**
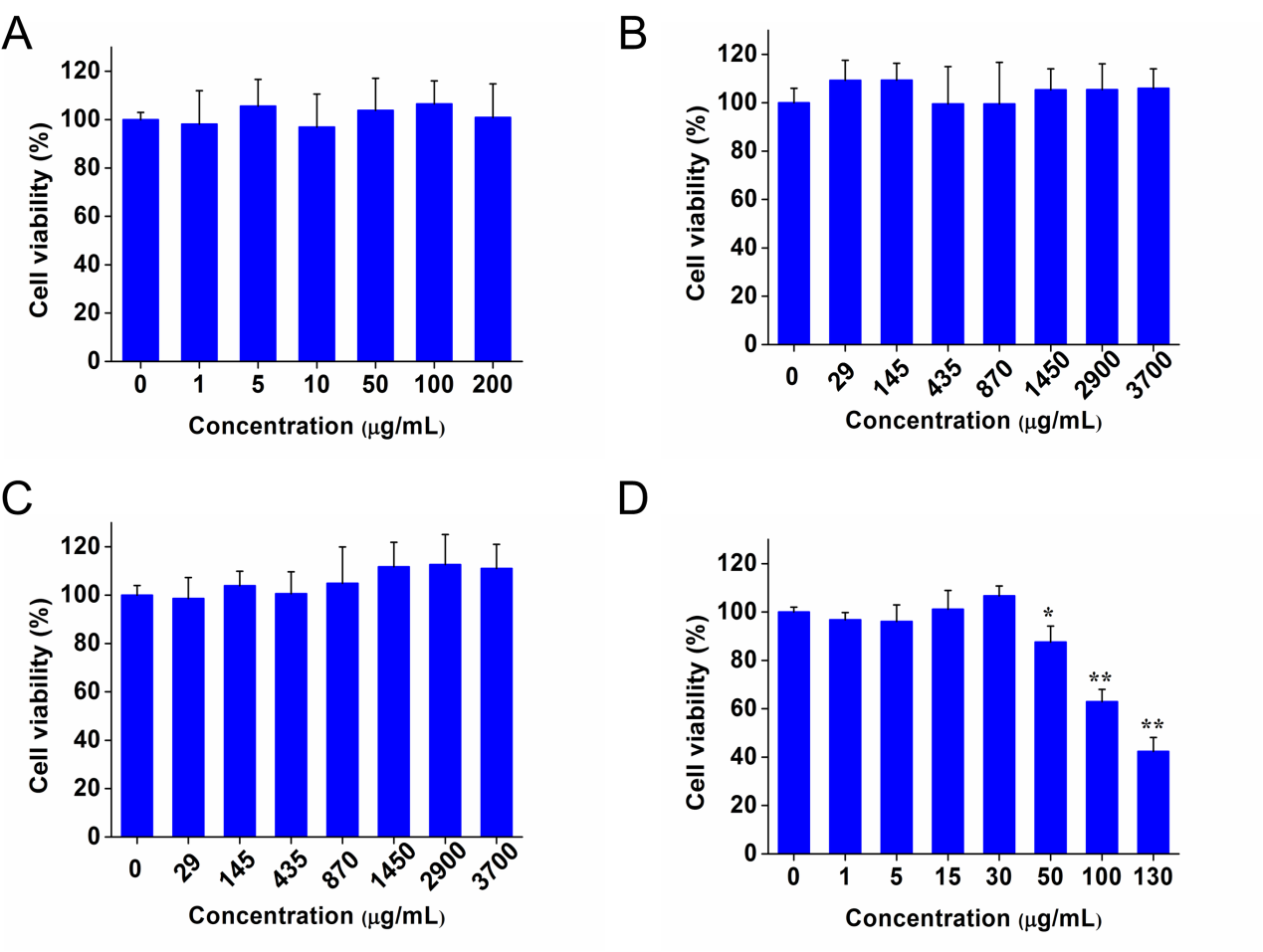
**

**Additional file 1: Figure S1.** The cytotoxicity of CsA (A), blank nanoparticle @PLGA-PEG (B), blank nanoparticle @PLGA-PEG-SS31 (C) and CsA@PLGA-PEG-SS31 (D) on H9C2 cells. ^∗∗^p<0.01 versus 0 group, ^∗^p<0.05 versus 0 group.

**
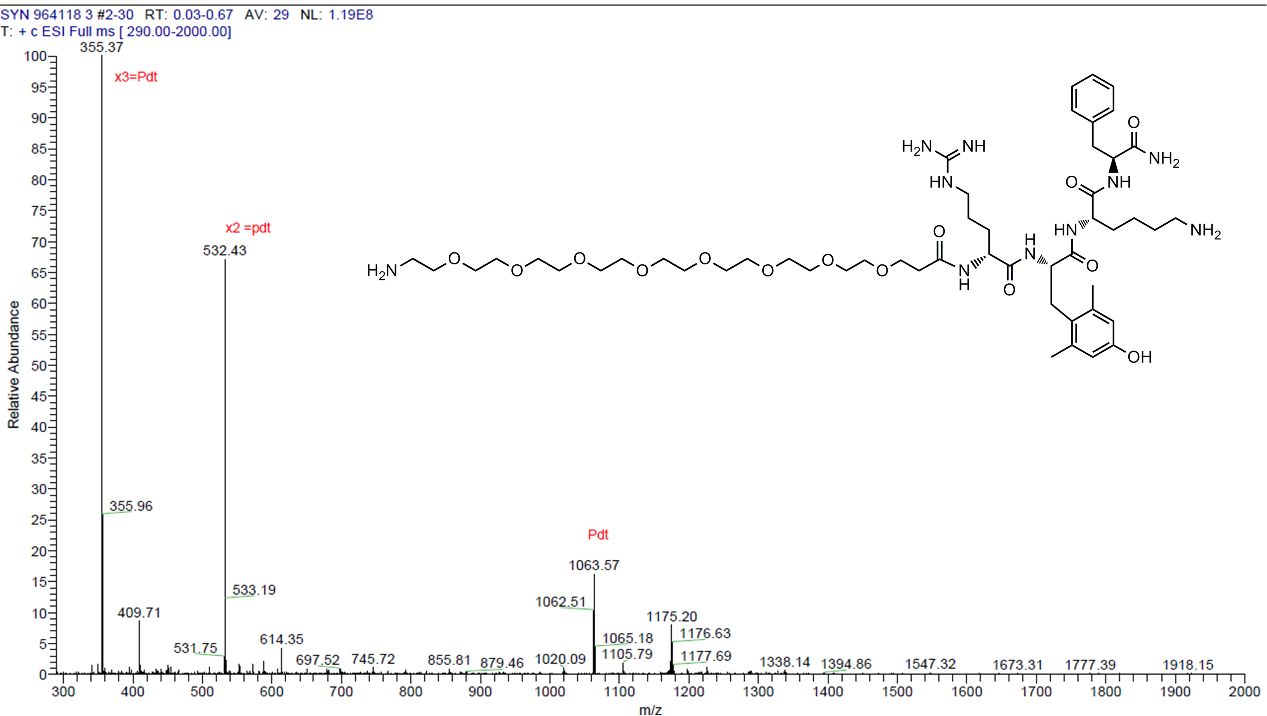
**

**Additional file 1: Figure** S2. The mass spectrum of SS31-PEG8.

**
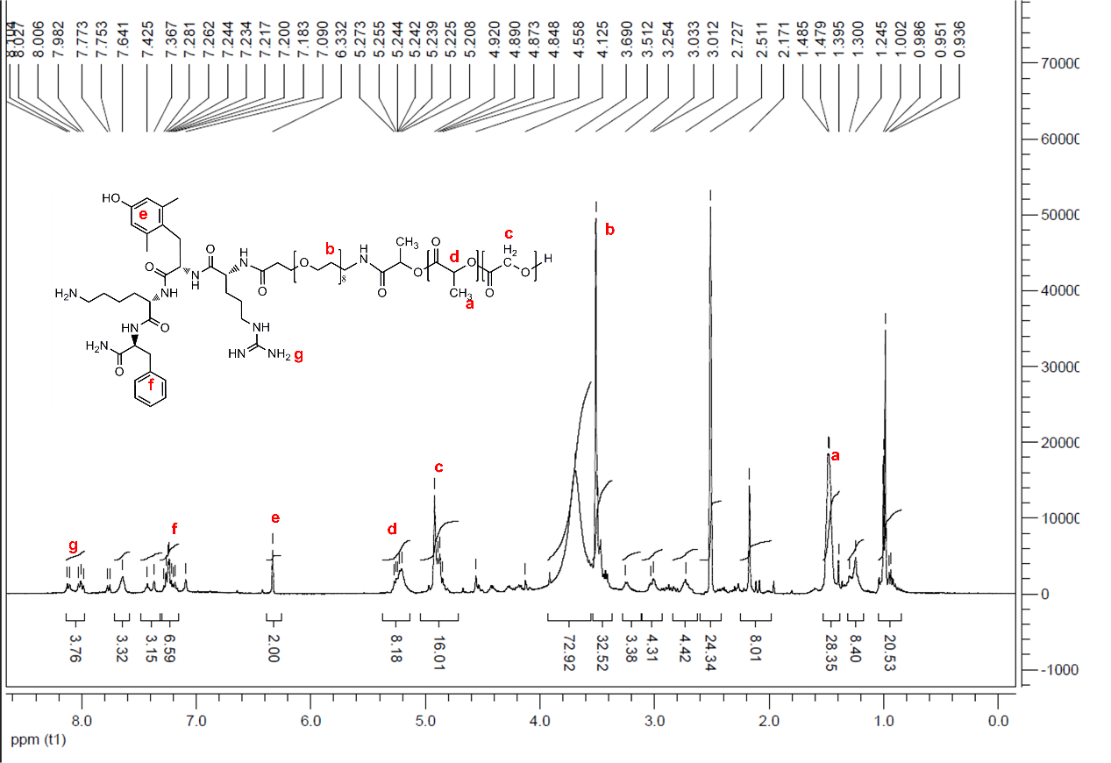
**

**Additional file 1: Figure** S3. The ^1^HNMRspectrum of PLGA-PEG-SS31.

**Additional file 1: Table S1.** The hemolysis rate (HR) of blank nanoparticles on rat red cells

|  | @PLGA-PEG | |  | @PLGA-PEG-SS31 | |
| --- | --- | --- | --- | --- | --- |
|  | 1 mg/mL | 2 mg/mL |  | 1 mg/mL | 2 mg/mL |
| HR(%) | 1.0 ±0.3 | 1.9 ±0.5 |  | 1.0 ±0.2 | 2.4 ±0.4 |
